# Progress on Physical Processes for Boiler Feedwater Deoxygenation

**DOI:** 10.3390/membranes16070244

**Published:** 2026-07-17

**Authors:** Binglin Li, Andong Jian, Jiayu Lu, Jiaxi Lv

**Affiliations:** State Grid Shaanxi Electric Power Co., Ltd., Ultra-High Voltage Company, Xi’an 710024, China; j1224345211@outlook.com (A.J.); lujiayu972501@outlook.com (J.L.); lvjiaxi124@outlook.com (J.L.)

**Keywords:** boiler feedwater, dissolved oxygen, membrane deoxygenation, mass transfer enhancement, hollow fiber membrane

## Abstract

With the expansion of thermal power units and higher operating parameters, oxygen corrosion in boiler feedwater has become increasingly serious. Traditional methods such as thermal, stripping, and vacuum deaeration are mature but limited by energy consumption, system complexity, and adaptability. In contrast, membrane deaeration has emerged as a promising alternative due to its energy efficiency, compact design, and superior adaptability. Recent advances in membrane materials and module configurations have enabled efficient dissolved oxygen removal to ppb levels under mild operating conditions. This review summarizes the mechanisms, equipment, and application features of conventional methods, and outlines progress in membrane deaeration regarding mass transfer, module design, and process integration. Future development will focus on multi-technology coupling, mass transfer intensification, and intelligent control to achieve ppb-level oxygen removal.

## 1. Introduction

Thermal power generation plays a vital role in the global power system [1,2,3]. As the core equipment in thermal power plants, boilers are responsible for converting the chemical energy of fuel into thermal energy, thereby directly influencing the stability and quality of power supply [4,5]. During long-term operation, boilers and their feedwater systems are exposed to complex hydro-chemical environments and harsh service conditions, making corrosion an inevitable issue [6]. Corrosion has become one of the key factors affecting the safety, reliability, and service life of power plant equipment. From a mechanistic perspective, the primary forms of boiler corrosion include oxygen corrosion [7], acid corrosion [8], and stress corrosion [9]. Among these, oxygen corrosion caused by dissolved oxygen (DO) is particularly prevalent in practical operation. Dissolved oxygen refers to molecular oxygen present in water, and under normal temperature and pressure conditions, its concentration can reach 7–9 mg/L [10]. At elevated temperatures and pressures, dissolved oxygen exhibits strong electrochemical corrosivity toward metallic materials. It accelerates corrosion through direct oxidation [11], electrochemical reactions [12], and differential aeration mechanisms [13], resulting in the formation of iron oxides that reduce heat transfer efficiency. In severe cases, this can lead to water wall corrosion or even tube rupture due to overheating, posing significant risks to boiler safety [14]. To avoid such risks, industry organizations such as the American Society of Mechanical Engineers and the American Boiler Manufacturers Association recommend that the dissolved oxygen content in boiler feedwater be maintained below 7 μg/L to ensure long-term safe operation [15].

Conventional deoxygenation methods can be broadly classified into chemical and physical methods. Although chemical methods are effective in reducing dissolved oxygen [16,17,18,19], they present several drawbacks, including reagent toxicity (e.g., hydrazine [20]) and the accumulation of byproducts (e.g., increased dissolved solids from sodium sulfite [21]). In contrast, physical methods have gained increasing attention due to significant advantages, such as the absence of chemical additives [22], elimination of secondary pollution risks [6], and lower maintenance requirements [23]. In particular, for high-pressure boiler systems, physical methods, such as thermal deaeration [24], vacuum deaeration [23], and membrane separation [25], can meet stringent deoxygenation standards while reducing energy consumption and environmental pollution. This review focuses on physical deoxygenation technologies for boiler feedwater, systematically summarizing traditional methods and recent research progress, with the aim of providing theoretical support and practical guidance for the optimization and innovation of boiler deoxygenation systems. This article first outlines the fundamental mechanisms and performance of conventional thermal, stripping, and vacuum deoxygenation methods. It then provides a comprehensive analysis of the emerging membrane deoxygenation technology, detailing advancements in mass transfer mechanisms, module design, and material selection. Finally, the review discusses future development trends, including multi-technology coupling and intelligent control, to achieve highly efficient and in-depth deoxygenation.

## 2. Traditional Physical Methods

**Figure 1:**
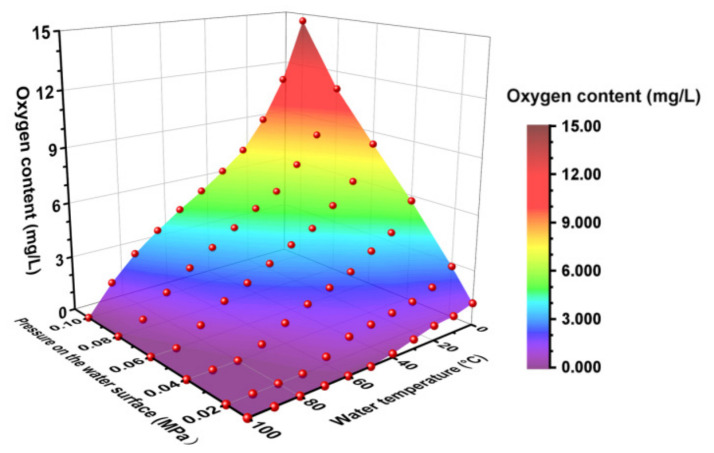
Saturated DO concentration in pure water vs. temperature and pressure.

The corrosion of boilers caused by dissolved oxygen is essentially an electrochemical process occurring between metals (primarily iron) and oxygen. The corresponding anodic and cathodic reactions of the galvanic cell can be expressed as follows in Table 1:

Here, $E^{{}^{\circ}}$ represents the standard electrode potential of the half-cell reaction. Due to the relatively high positive potential of oxygen reduction, dissolved oxygen can readily react with metals and accelerate corrosion, as shown in Table 1. Therefore, controlling and reducing oxygen concentration is an effective strategy to limit its corrosive effects on steel. Physical deoxygenation methods primarily remove dissolved oxygen by altering its solubility in water. According to Henry’s Law [26], at a constant temperature, the concentration of a gas dissolved in a given volume of liquid is directly proportional to its partial pressure at the gas–liquid interface. This relationship can be expressed as follows:

(1) $$C_{O_{2}}\;=\;k_{H}\;\times {\;P}_{O_{2}}$$

where $C_{O_{2}}$ represents the concentration of dissolved oxygen in water,${\;P}_{O_{2}}$ represents the partial pressure of oxygen in the gas phase, and $k_{H}$ represents Henry’s constant. Furthermore, the higher the water temperature, the lower the solubility of gases in the water. When the water temperature reaches its boiling point, the vapor pressure above the water surface equals the external pressure, and the partial pressures of other gases are all zero, thus eliminating their ability to dissolve gases and causing various dissolved gases in the water to separate out. The relationship between the saturated dissolved oxygen content of boiler feedwater and pressure and temperature is shown in Figure 1.

According to the two-film theory, the transfer of dissolved oxygen from the liquid phase to the gas phase is jointly controlled by liquid film resistance and gas film resistance. In traditional physical deoxygenation processes, the mass transfer rate can be expressed as [27]:

(2) $$N\;=\;K_{L}\;\times \;a\;\times \;(C\;-\;C^{*})$$

where *N* represents the oxygen mass transfer flux, $K_{L}$ represents the liquid phase mass transfer coefficient, *a* represents the gas–liquid contact area per unit volume, *C* represents the dissolved oxygen concentration in the liquid phase, and C* represents the dissolved oxygen concentration in equilibrium with the gas phase. Therefore, mass transfer enhancement can be achieved through three pathways [28,29,30]: (1) increasing the specific surface area, such as using atomized spraying or packed towers; (2) improving the mass transfer coefficient, such as introducing turbulence or hyper-gravity fields; and (3) increasing the mass transfer driving force (*C* − C*), such as reducing the partial pressure of oxygen in the gas phase or increasing the water temperature. Traditional thermal deoxygenation, analytical deoxygenation, and vacuum deoxygenation technologies all optimize processes around the above pathways, but due to limitations in macroscopic equipment structure and operating conditions, the mass transfer enhancement effect has a theoretical upper limit.

### 2.1. Thermal Deoxygenation

**Figure 2:**
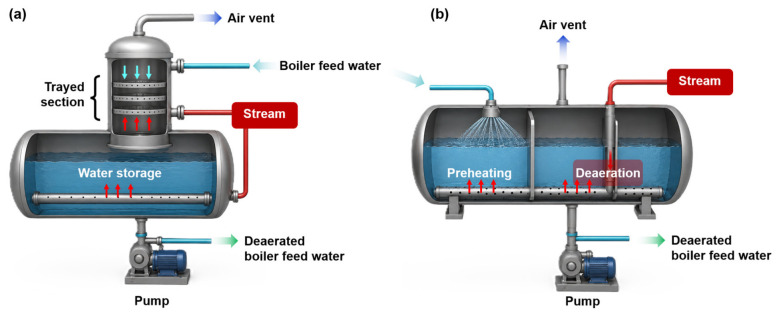
The schematic diagram of (**a**) typical horizontal tray-type deaerator and (**b**) typical spray-type deaerator.

Thermal deoxygenation can be classified into atmospheric thermal deoxygenation and jet-type thermal deoxygenation, as shown in Figure 2. The principle involves heating boiler feedwater to its boiling point, thereby reducing the solubility of dissolved oxygen and promoting its continuous release from the liquid phase. The liberated oxygen, together with water vapor, is subsequently removed from the system.

In atmospheric thermal deoxygenation, the deaerator operates at a pressure slightly above atmospheric pressure (typically around 20 kPa gauge), corresponding to a boiling temperature of approximately 104 °C. Under these conditions, dissolved oxygen and other non-condensable gases are rapidly released and discharged through continuous venting, achieving effective deoxygenation. As the water temperature approaches its boiling point, the mass transfer resistance is significantly reduced, resulting in enhanced oxygen removal efficiency. From the perspective of mass transfer enhancement, advancements in thermal deoxygenation technologies primarily focus on two aspects [31]: first, increasing the gas–liquid contact area through optimization of structural and operational parameters, such as tray opening ratio and spray density; second, reducing liquid film thickness and associated mass transfer resistance by leveraging the turbulence induced by steam injection or jet effects. The steam–water contact process enhancement scheme proposed by Istre et al. [32] essentially improves the overall mass transfer coefficient by improving the uniformity of the two-phase flow distribution. Santoro et al. [33] proposed a scheme to improve the internal gas–liquid mass transfer efficiency from the perspective of tray configuration and support structure optimization. They enhanced the contact uniformity between steam and liquid phases through microstructure design, thereby optimizing the overall deoxygenation performance. Wolf et al. [34] optimized the thermal contact path between steam and feedwater by introducing multi-stage mass transfer zones and different cross-section packing designs inside the deaerator. At the same time, they used a heat-exchange heat medium to preheat feedwater to improve deoxygenation efficiency and enhance the stability and maintainability of the equipment under varying operating conditions.

Jet-type thermal deoxygenation typically utilizes steam jetting devices to create a negative pressure environment inside the deaerator, rapidly heating the feedwater and ensuring full contact with steam, thereby promoting the release and removal of dissolved oxygen [35]. Under the jetting action, water is atomized or forms a thin liquid film, significantly increasing the gas–liquid contact area and accelerating the rate of oxygen transfer from the aqueous phase to the gas phase, thus achieving rapid removal of dissolved oxygen in a short time. Compared to atmospheric deaerators, spray structures in industrial boilers and power units can maintain stable, low oxygen content under medium-to-high flow conditions, demonstrating excellent dynamic adaptability and high-efficiency deaeration performance. Burke and co-workers [36] proposed a nozzle assembly design for a spring-driven jet diffuser that can form an annular jet orifice according to the feedwater pressure to achieve uniform spray distribution. This design not only improves the water atomization quality and spray pattern stability but also maintains high-efficiency deoxygenation performance over a wide flow range. They [37] further optimized the nozzle assembly design, realizing tool-free installation and disassembly and reducing the difficulty of equipment maintenance, while ensuring the stability of the spray effect and deoxygenation efficiency. In addition, Averesch [38] proposed connecting the nozzle unit to the deaerator body through a flange to achieve modular installation and consistent spray geometry, thereby ensuring uniform contact between water flow and steam in the entire deaerator volume and improving gas–liquid mass transfer efficiency. However, the mass transfer enhancement of thermal deoxygenation is limited by the physical boundary of boiling heat transfer, and the mass transfer driving force is significantly reduced under low temperature conditions, making it difficult to meet the stringent requirements of supercritical units for dissolved oxygen.

A comparison of thermal deaerators and outlet dissolved oxygen performance are shown in Table 2. A clear trend is observed in terms of oxygen removal efficiency: conventional rotating film deaerators achieve outlet dissolved oxygen levels in the range of 6–10 μg/L, whereas thermally driven and nitrogen-assisted systems can reduce oxygen concentrations to below 7 ppb under optimized conditions. The improvement in performance is primarily attributed to enhanced gas–liquid interfacial area (rotating film and spray structures), reduced oxygen partial pressure (nitrogen sweep and vacuum-assisted designs), and intensified mass transfer driven by thermal equilibrium effects. In addition, system-level integration strategies, such as re-oxygenation suppression using closed surge tanks, further improve overall oxygen control stability.

### 2.2. Stripping/Desorption Deoxygenation

Stripping deoxygenation involves intensely mixing an oxygen-free inert gas with the water to be deoxygenated. This reduces the partial pressure of oxygen in the gas phase at the liquid–gas interface, lowering the equilibrium concentration of dissolved oxygen in the liquid phase. This promotes the diffusion of dissolved oxygen from the liquid phase to the gas phase, thus reducing the oxygen content of the feedwater. The diffused oxygen flows with the inert gas to the reactor, where it reacts with charcoal to convert into carbon dioxide. This carbon dioxide is then used as an oxygen-free gas and intensely mixed with the water to be deoxygenated, and the cycle continues [46].

Zhu et al. [47] proposed a low-temperature deoxygenation system design that uses catalytic deoxygenators to replace traditional electric heating charcoal furnaces in order to reduce dependence on heat sources and improve operating conditions; however, CO_2_ is also an important factor leading to carbonic acid corrosion. Due to the solubility of CO_2_ in water, the pH value of water is reduced by about 0.2 to 0.3, which in turn enhances the dissolved oxygen corrosion effect and is detrimental to the safe operation of boilers and feedwater systems. Since conventional deaerators alone cannot completely eliminate all CO_2_, Ding et al. [48] proposed a room-temperature hydrogen deoxygenation method to eliminate carbonic acid corrosion caused by CO_2_. However, since hydrogen has higher industrial application value, Jin et al. [49] proposed using carbon molecular sieve adsorption columns to adsorb adsorbable gases in feedwater under vacuum conditions. This method can reduce the content of dissolved gases, including oxygen and CO_2_, in continuous operation, but once activated carbon is saturated, the operation of the reactor becomes unstable, leading to a decrease in deoxygenation performance. Against this backdrop, the application of high gravity/rotating packed bed (RPB) technology in oxygen stripping and CO_2_ adsorption provides a potential means of mass transfer enhancement for desorption. Yuan et al. [50] reported that in the application of jet fuel deoxygenation, RPB can significantly improve the gas–liquid interface area and mass transfer rate through a high-gravity field, and rapidly reduce the dissolved oxygen in the fuel to less than 1 ppm at a rotation speed of 1200 rpm, while the device volume is only about 0.02 m^3^. Although these designs have alleviated the constraints of the original desorption deoxygenation device to some extent, the related technologies still face problems such as easy saturation of reactants, high dependence on temperature control, and still-large energy consumption and maintenance costs. Therefore, desorption deoxygenation has been gradually replaced by other deoxygenation processes in modern boiler feedwater treatment systems.

### 2.3. Vacuum Deoxygenation

Vacuum deoxygenation utilizes the fact that water boils at a lower temperature under vacuum than at normal pressure, thereby reducing the solubility of oxygen to near zero at low to medium temperatures (30–60 °C) to achieve deoxygenation [51]. As early as the 1960s and 1970s, the performance verification and improvement exploration of the system began. Adams [52] reported that the vacuum deoxygenation system could reduce the dissolved oxygen in the Pan American Petroleum Corp. water injection field from about 9 ppm to 0.5 ppm and operate stably for a long time at low operating costs. Kogure [53] used a vacuum spray column to improve gas–liquid contact and optimize the operating pressure range to enhance the deoxygenation effect. In conjunction with the deoxygenation method, he proposed a scheme to reduce the dissolved oxygen in pure water to <10 ppb by spray packing and inert gas in combination under conditions where the vacuum degree is slightly higher than the saturated vapor pressure. Zhao et al. [46] combined vacuum degassing with vacuum N_2_ stripping coupling technology to enhance gas–liquid mass transfer and deoxygenation effects. The results show that under the conditions of coupled vacuum suction and nitrogen stripping, the overall dissolved oxygen removal efficiency η reached 97.34%, which is significantly improved compared with single vacuum degassing (η = 89.95%). Jun et al. [54] proposed a vacuum degassing system based on the Venturi cavitation vacuum bubble method. By adjusting the vacuum degree, a dissolved oxygen level close to 0 mg/L was achieved for 360–400 L drinking water samples at a vacuum degree of about 1 kPa. Compared with thermal deoxygenation technology, the heating conditions of vacuum deoxygenation method are improved and the boiler self-consumption of steam is reduced. However, the requirements for key equipment such as jet pumps and pressurization pumps are higher than those for thermal deoxygenation, and the cost of heat-exchange equipment and circulating water tank is also increased. Chu et al. [55] proposed a liquid deoxygenation system that integrates hyper-gravity equipment, a vacuum device, and a gas supply module. This system can use hyper-gravity-enhanced degassing, vacuum suction deoxygenation and inert gas desorption deoxygenation separately or simultaneously according to different working conditions, so as to realize the free switching and synergistic optimization of multiple deoxygenation methods.

## 3. Novel Deoxygenation Membrane Method

With the advancement of membrane materials science and separation technology, membrane deoxygenation technology has gradually emerged since the 1990s. Its earliest origin was in the application scenario of oxygenating blood via the exploration of gas–liquid transfer in microporous membranes [56]. In the early exploration work of Yang and Cussler [57], inert gas and oxygen were used as research objects. By constructing hollow fiber membrane modules, the key mass transfer mechanism controlling degassing efficiency was revealed, laying a key theoretical foundation for the industrial application of membrane deoxygenation. Subsequently, the application of membrane deoxygenation technology to several key industrial scenarios was attempted, including supplying oxygen to closed subsea facilities [58] and deep deoxygenation of ultrapure water for the semiconductor [59] and energy industries [60], and showed significant application potential. Compared with traditional technologies, membrane deoxygenation has achieved large-scale industrial application due to its advantages such as room-temperature operation, low energy consumption, no chemical addition, and modular design. Table 3 compares major dissolved oxygen removal technologies, including thermal, stripping/desorption, vacuum, membrane, and catalytic membrane processes, in terms of operating temperature, achievable DO level, energy demand, and key advantages and limitations. Overall, thermal and stripping-based methods are mature and widely used but generally require higher energy input or carrier gas assistance, whereas membrane and catalytic membrane technologies operate under milder conditions and offer lower energy consumption with higher separation efficiency, though they are still limited by cost and long-term stability.

### 3.1. Membrane Deoxygenation Mechanism

Membrane deoxygenation is essentially a physical separation process, and its theoretical basis still follows Henry’s Law. The core innovation lies in its optimization of the gas–liquid mass transfer process, decoupling fluid flow from interphase mass transfer and thus improving the efficiency of physical deoxygenation, with the selective permeability of the membrane material serving as a key enabling factor [61,62]. In the deoxygenation process of boiler feedwater, the membrane material, acting as an inert medium, isolates the aqueous phase from the gas phase [63]. Water molecules are blocked due to the hydrophobic properties of the membrane material, while dissolved oxygen molecules diffuse from the liquid phase side to the low-pressure or purge gas side driven by the partial pressure difference, thereby achieving the removal of dissolved oxygen from the boiler feedwater. The diffusion behavior of dissolved oxygen between gas and liquid can be described by Fick’s First Law:

(3) $$\frac{\mathrm{dm}}{\mathrm{dt}}\;=\;-\mathrm{DA}\frac{\mathrm{dc}}{\mathrm{dx}}$$

where dm/dt is the oxygen mass transfer rate, D is the oxygen diffusion coefficient, A is the effective mass transfer area, and dc/dx is the oxygen concentration gradient. The rate of dissolved oxygen diffusion in water is positively correlated with the gas–liquid contact area and the oxygen concentration gradient. Therefore, it is necessary to maximize the gas–liquid contact area of the membrane material and artificially reduce the oxygen partial pressure on the other side of the membrane. Equation (4) describes the overall mass transfer resistance to identify the rate-limiting step in the membrane deoxygenation process [64].

(4) $$\frac{1}{K}=\frac{1}{k_{l}}+\frac{1}{k_{m}}+\frac{1}{k_{g}}$$

where 1/kL, 1/k_m_ and 1/k_e_ represent the layer resistance of the liquid boundary, membrane and gas boundary, respectively. Based on Fick’s Law and the boundary layer theory, the liquid and gas phase mass transfer coefficients (k_l_ and k_e_) are significantly influenced by hydrodynamic conditions, as flow velocity dictates the boundary layer thickness. As shown in Equation (5), the membrane mass transfer coefficient (k_m_) is governed by the membrane’s structural properties and gas diffusivity, expressed as [65]:

(5) $$k_{m}\;=\;\frac{D_{m}\varepsilon }{\tau \delta }$$

where D_m_ represents the diffusion coefficient of the gas within the membrane pores, ϵ is the membrane porosity, τ denotes the pore tortuosity, and δ is the membrane thickness. Compared to traditional absorption processes, in membrane deoxygenation technology, the gas and liquid phases flow independently on both sides of the membrane. Because no phase mixing occurs, problems such as mist entrainment and flooding, common in traditional absorption processes, are effectively avoided. Furthermore, the membrane, as a self-supporting structure, eliminates the need for additional supports, greatly simplifying the complexity of assembling membrane modules [66,67]. By constructing membrane modules through modular design, a gas–liquid contact area far exceeding that of traditional methods can be achieved. When this interface works in conjunction with a vacuum system, the driving force for dissolved oxygen separation can be significantly enhanced, thus exhibiting higher process efficiency [68].

**Figure 3:**
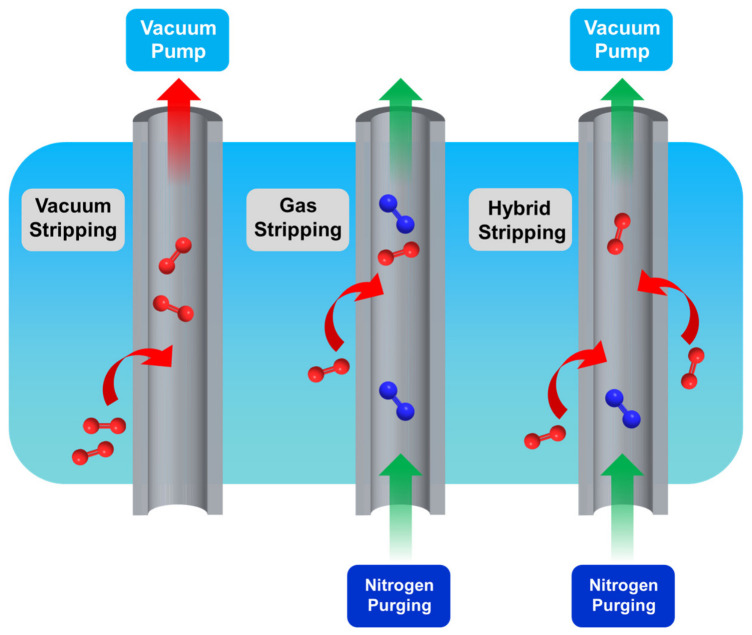
Three membrane degassing desorption methods.

As shown in Figure 3, in order to maintain a low oxygen partial pressure at the membrane interface to enhance the driving force of mass transfer, the following three desorption methods are mainly used in practice [69]: (1) Vacuum desorption: By establishing a vacuum environment in the opposite membrane cavity, the total pressure of the gas phase is reduced, thereby reducing the oxygen partial pressure and forming the driving force for the transfer of dissolved oxygen across the membrane. This method does not require the introduction of external gas and the process is clean, but a vacuum pump system is required. (2) Purge degassing: An inert gas (such as nitrogen) is introduced into the purge side of the membrane module. The oxygen partial pressure in the gas phase on this side is reduced by dilution. This method operates at a pressure close to atmospheric pressure and the equipment requirements are relatively simple, but it consumes purge gas. (3) Composite desorption: This method combines vacuum desorption and purge desorption. Vacuum suction is applied while purge gas is introduced, which can reduce the oxygen concentration by gas dilution and further reduce the total pressure by vacuum, thereby forming a superposition effect of a stronger partial-pressure difference driving force, which can significantly improve deoxygenation efficiency and operational flexibility.

Membrane materials can be mainly classified into porous membranes and dense membranes according to their microstructure. The two have significant differences in separation mechanism and application scenarios. The separation process of porous membranes mainly depends on the micro–nano-scale pore structure inside. The porous membranes provide a gas-filled hydrophobic interface through which oxygen transfers according to partial-pressure difference, with membrane wetting and liquid entry pressure (LEP) being key practical constraints. When a mixed gas or liquid encounters the membrane surface, the components with molecular size smaller than the membrane pore size can pass through the pores, while the components with larger size are trapped, thus achieving separation. Depending on the dominant mass transfer mechanism, its separation process can be manifested in various forms such as Knudsen diffusion, viscous flow, and surface diffusion. Membrane wetting significantly deteriorates degassing efficiency. The core mechanism involves an increase in mass transfer resistance. Specifically, a higher wetting constant corresponds to a lower membrane-phase mass transfer coefficient, which subsequently reduces the overall mass transfer coefficient and ultimately diminishes the stripping flux, thereby limiting process performance [70]. Rangwala [71] found that even marginal (≲2%) local pore wetting can raise the membrane-phase resistance so that it accounts for up to ~60% of the total mass transfer resistance, and the effective overall mass transfer coefficient drops accordingly, whereas a more extensively wetted state can push the membrane resistance ∼two orders of magnitude above the non-wetted baseline. Nakhjiri and Heydarinasab [72] employed a mass transfer resistance-in-series model to investigate the CO_2_ transport process within a hollow fiber membrane contactor (HFMC). Their comprehensive modeling and CFD simulation demonstrated that membrane wetting along the fiber axis introduces significant additional resistance to mass transfer. This wetting phenomenon directly increases the membrane-phase resistance term in the overall mass transfer coefficient equation, thereby substantially reducing the overall mass transfer coefficient and, consequently, the gas stripping efficiency. Toh et al. [73] demonstrated that the hydrophobicity of PVDF-HFP membranes can be effectively tuned by optimizing the polymer concentration, using ethanol as a coagulation medium, and incorporating modified silica nanoparticles. These adjustments enhanced the water contact angle from 100.4° to 163.1° and increased the LEP from 0.23 bar to 3.38 bar, thereby improving wetting resistance.

In contrast, the separation process of dense membranes follows the dissolution-diffusion mechanism [74]. This theory holds that gas molecules first dissolve on the surface of the dense membrane material and then diffuse under the drive of the concentration gradient or chemical potential difference in the membrane and finally desorb on the other side of the membrane. This process is closely related to the solubility and diffusion coefficients of the components in the membrane material. Therefore, its separation efficiency depends on the specific physicochemical interactions between the membrane material and the components to be separated, rather than simple size exclusion. This allows dense membranes to exhibit extremely high separation selectivity. However, in large-scale industrial water treatment scenarios such as boiler feedwater deoxygenation, which require efficient and low-cost removal of dissolved oxygen, hydrophobic porous membranes offer greater engineering advantages than dense membranes. These membranes enable gas transfer across a hydrophobic, gas-filled membrane interface, driven by an oxygen partial-pressure gradient. Their main benefits include high permeate flux, relatively mature fabrication technology, and cost-controllable production, making them a more practical and widely adopted choice [75,76,77].

### 3.2. Selection of Membrane Modules

**Figure 4:**
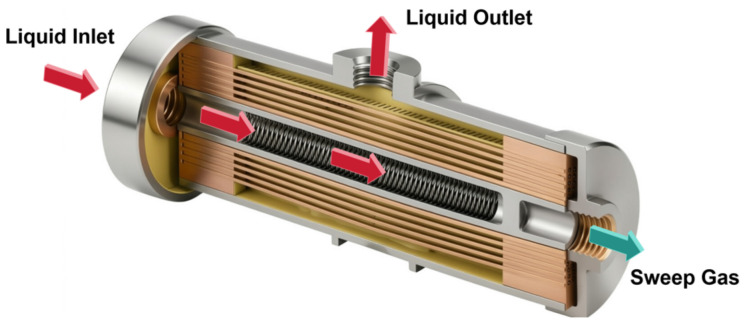
Classic design of membrane deoxygenation module based on polypropylene hollow fiber membrane.

In the design of membrane separation systems for boiler feedwater deoxygenation, the selection of membrane modules directly affects deoxygenation efficiency, operating energy consumption and engineering feasibility. Common membrane module forms in industry include plate and frame, spiral wound, tubular and hollow fiber [78,79]. The plate and frame structure is easy to maintain, but has low packing density and is prone to forming uneven flow areas; spiral wound modules have higher packing density, but complex flow channels, which limit the full utilization of their mass transfer area and have general anti-fouling performance; tubular modules have smooth flow channels and strong anti-fouling ability, but their mass transfer area per unit volume is usually low, and a larger equipment volume is often required to achieve the same treatment capacity. In comparison, hollow fiber membrane modules show significant advantages in boiler water deoxygenation applications. As shown in Figure 4, its core characteristic is that it can provide an extremely high specific surface area in a compact modular form, thereby greatly improving the gas–liquid mass transfer rate and meeting the process requirements of continuous and efficient deoxygenation. At the same time, the hydrophobic porous membrane materials such as polypropylene and polyvinylidene fluoride used in these modules [80] can effectively block liquid water permeation, ensuring the stability and reliability of long-term operation.

**Figure 5:**
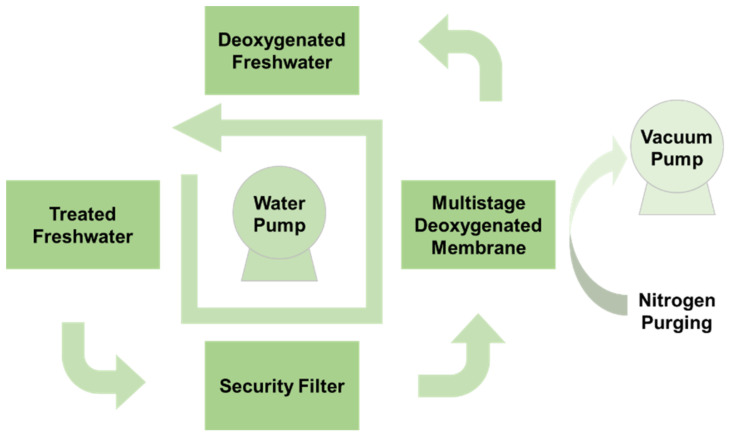
Classic boiler water deoxygenation process.

The typical boiler water membrane deoxygenation process is shown in Figure 5. To ensure long-term stable operation of the degassing membrane and prevent membrane pore blockage, the feedwater must be purified by sand filtration, activated carbon adsorption, reverse osmosis membrane desalination, and electrodialysis for deep deionization. This purified water first passes through a security filter to prevent membrane fouling, then enters the degassing membrane system. In the system, the water flows through multiple membrane modules connected in series, and dissolved oxygen is removed from the water under the combined action of vacuum and nitrogen purging provided by the system. The deoxygenated effluent can be used for subsequent processes, such as heating it in a heat exchanger and using it as boiler feedwater. The removed oxygen and purging nitrogen are finally extracted from the system by a vacuum pump.

### 3.3. Membrane Materials and Structural Design

The selection of membrane material is a critical factor influencing deoxygenation efficiency. A comparison of different membrane materials is shown in Table 4. Polypropylene (PP) was widely used in early applications due to its excellent chemical stability and cost effectiveness. However, its inherent hydrophobicity limitations or relatively low surface hydrophobicity restrict its deoxygenation performance. Chen et al. [81] prepared a polydimethylsiloxane/polypropylene (PDMS/PP) composite membrane by the dip coating method for efficient removal of dissolved oxygen in water. They found that multilayer coating can significantly improve the operation stability (>1760 min), while the deoxygenation rate and mass transfer coefficient only decreased slightly (about 3.8%), which proved that the composite membrane has both high efficiency and long-term stability in the deoxygenation process of boiler feedwater. Han et al. [82] experimentally studied the intrinsic oxygen mass transfer characteristics of a hydrophobic PP hollow fiber membrane. They found that the mass transfer coefficient does not increase monotonically with the increase in water flux, but increases significantly with the increase in air inlet pressure. Based on this, they proposed a new mass transfer model that combines liquid membrane renewal time and solubility equilibrium time, which shows that the total mass transfer rate is controlled by both interface renewal and oxygen diffusion rate. Peng et al. [83] obtained woven PP hollow fiber membranes by melt spinning and used them for membrane deoxygenation on a medium scale. The results showed that the woven type achieved a deoxygenation efficiency of 97.5% under vacuum conditions due to its transverse flow design, and the mass transfer coefficient was higher than that of the traditional fiber bundle packing structure. This further explored the industrial scale-up potential of PP materials for membrane deoxygenation.

**Figure 6:**
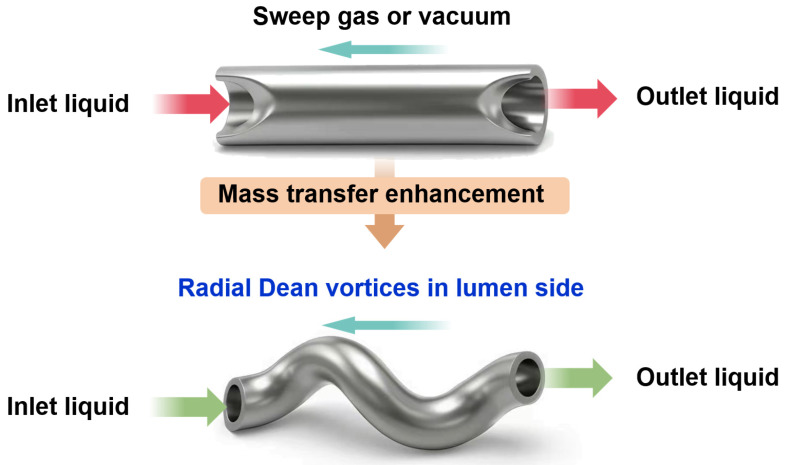
Mass transfer enhancement effect in membrane gas transfer process.

Polyvinylidene fluoride (PVDF) membranes have become another important choice due to their excellent corrosion resistance and thermal stability. Ci et al. [84] combined PVDF porous membranes with vacuum distillation process. Under the optimal conditions of a 0.1 MPa vacuum degree, 30 °C temperature, and 160 L/h flow rate, the dissolved oxygen removal rate reached up to 94.8%, which proved the advantages of this technology in terms of efficient deoxygenation and process controllability. Rahbari-Sisakht et al. [85] improved the hydrophobicity of PVDF hollow fiber membranes by adding modified macromolecules to the surface of the membrane. When the SMM concentration increased to 6 wt.%, the CO_2_ desorption flux and efficiency in water were the highest, which also showed that increasing the liquid temperature can significantly increase the gas flux. Using non-porous hollow fiber membranes for feedwater deoxygenation is also feasible. Ito et al. [86] studied the process of using silicone rubber as a hollow fiber membrane for pure water deoxygenation. The results showed that during vacuum degassing, when the pressure on the permeate side dropped to 13 kPa, the dissolved oxygen dropped from 6 mg/L to below 2 mg/L. In addition, the gas phase driving force theory developed based on gas permeability accurately predicted the influence of pressure and flow rate on deoxygenation efficiency, demonstrating the controllability of the technology. The structural design of the membrane module has an important influence on the mass transfer efficiency. Although traditional straight fiber membranes have a simple structure, they have the problem of insufficient mass transfer efficiency. Moulin et al. [87] found through pure water oxygenation experiments that the oxygen mass transfer coefficient of the spiral hollow fiber membrane was 2–4 times higher than that of the traditional straight fiber membrane, confirming that the Dean vortex can effectively destroy concentration polarization and enhance liquid phase mass transfer. Kaufhold et al. [88] further investigated the influence of Dean vortices on the oxygen transfer rate. They demonstrated that generating Dean vortices in curved hollow fiber membranes, specifically helical and meander-shaped geometries, enhanced the oxygen transfer rate by up to a factor of 2.4 compared to straight fibers. For helical fibers, the enhancement factor showed a linear dependence on the Dean number. For meander-shaped fibers, the relationship exhibited a slightly s-shaped dependency, with a critical Dean number range between 10 and 20, beyond which the mass transfer enhancement rapidly adjusted to levels comparable to helical fibers. Tepper et al. [89] developed helical-ridge hollow fiber membranes that induce secondary flow in the lumen, significantly enhancing gas–liquid mass transfer and achieving up to a 10-fold improvement in transmembrane gas fluxes. Kong et al. [90] focused on the mass transfer enhancement effect of the Dean vortex on pure water deoxygenation. They found that the deoxygenation rate of the spiral hollow fiber membrane was twice that of the traditional straight fiber membrane by inducing transverse fluid disturbance through a vortex, which significantly improved tube-side mass transfer, as shown in Figure 6.

**Figure 7:**
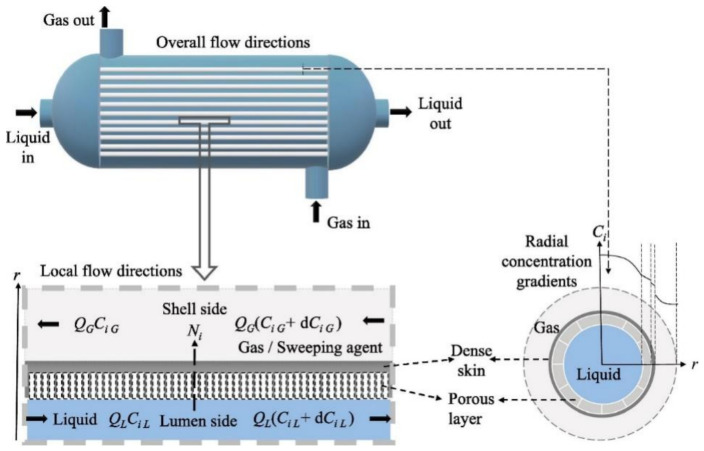
High-efficiency membrane contactor module and mass transfer gradient for in-cavity liquid flow in scavenging stripping. Obtained from [91].

Alhmiedy et al. [91] focused on the quantitative relationship between mass transfer efficiency and liquid velocity, membrane area and pressure drop when liquid flows in a tube. By establishing a simplified one-dimensional model for the size design of hollow fiber membrane degassing applications, they found that the mass transfer efficiency depends only on the ratio of liquid velocity to membrane area. For example, a fiber with an inner diameter of 140 μm can achieve an oxygen removal efficiency of 98% at a specific flow rate ratio, and the pressure drop can be accurately predicted by the Hagen–Poiseuille law, providing a reliable theoretical tool for the scale-up design of membrane deoxygenation, as shown in Figure 7.

In the field of boiler water treatment, membrane deoxygenation technology has shown significant advantages. Liu et al. [92] combined micro-interface broad-spectrum analysis technology with vacuum membrane deoxygenation in steam injection boiler water treatment and found that under the conditions of a 0.098 MPa vacuum degree and 20 °C water temperature, the oxygen content of feedwater was significantly reduced, which proved that the technology has promotion value. Xu et al. [93] studied the actual application efficiency of membrane deoxygenation technology in the vaporization cooling system of a steel rolling furnace and proved that the deoxygenation effect of the technology is good and stable (up to 1 ppb) and the investment recovery period is short (1.4 years), which strengthens its advantages in energy saving. Shao et al. [94] studied the vacuum deoxygenation process of boiler feedwater through a pilot-scale hollow fiber membrane. Experiments showed that the deoxygenation efficiency was significant under the conditions of a flow rate of 4.9 m^3^/h and a vacuum degree of −0.095 MPa, but the use of surface water caused membrane fouling (composite scale of organic matter and aluminum silicate). After air backwashing, the efficiency recovered to 80%, which verified the high efficiency and controllable pollution of the technology.

In addition to the high oxygen removal efficiency demonstrated in pilot-scale studies, long-term operational stability remains a critical barrier for the industrial application of membrane deaeration in boiler feedwater systems. Membrane fouling is a significant challenge that deteriorates membrane performance by reducing flux and selectivity. To mitigate fouling, strategies such as optimizing hydrodynamic conditions to enhance surface shear and modifying membrane surface properties for improved anti-fouling characteristics are effective [95]. Shao et al. [94] also reported that membrane modules operating under pilot-scale conditions suffered from performance decline due to the accumulation of composite foulants, including organic matter and inorganic species such as aluminum silicates. Similar observations have been reported by Horseman et al. [96], who found that membrane fouling typically evolves from reversible deposition in the early stage to more compact and strongly adhered layers during long-term operation, resulting in increased mass transfer resistance and reduced deoxygenation efficiency. Although physical cleaning methods such as air backwashing can partially restore performance, repeated fouling–cleaning cycles may still lead to gradual performance degradation over extended operation periods [97]. Li et al. [98] revealed that Dean vortices, induced by specific flow configurations, can substantially alleviate membrane fouling. The vortices produce strong shear stresses near the membrane surface, which continuously remove accumulating particles and prevent the development of a compact cake layer, thereby helping to sustain stable permeate flux.

Membrane wetting is another major challenge affecting long-term stability. Ibrahim et al. [99] highlighted membrane wetting as one of the most significant obstacles to the industrial application of membrane contactors. Once liquid intrusion occurs into the hydrophobic membrane pores, the gas–liquid interface is disrupted, leading to a sharp decline in oxygen transfer efficiency. Wetting is often induced by the presence of surfactants, organic contaminants, or long-term fouling layers, which reduce the effective surface tension of the liquid phase. Chabanon et al. [100] demonstrated that even slight wetting of membrane pores can substantially increase mass transfer resistance and deteriorate separation performance. Therefore, recent studies have increasingly focused on developing superhydrophobic and omniphobic membranes to mitigate wetting risks and enhance the long-term operational reliability of membrane contactor systems. Membrane wetting leads to the loss of a stable gas–liquid interface within the membrane pores, which significantly deteriorates mass transfer efficiency and separation performance [101]. To address this limitation, surface modification strategies, including the construction of re-entrant surface structures and fluorination, have been widely explored. These approaches effectively increase the LEP, thereby delaying or suppressing pore wetting [102,103]. For instance, omniphobic membranes have demonstrated substantially higher LEP values (typically 1.5–5.5 bar) compared to conventional hydrophobic membranes (0.5–3.5 bar), along with excellent repellency toward low-surface-tension liquids containing surfactants or oils [104,105]. In gas–liquid membrane contactors for CO_2_ capture, superhydrophobic ceramic membranes have shown remarkable improvements, with water contact angles reaching up to 170.7° and CO_2_ capture efficiency increasing from 28.55% to 98.55% under laminar flow conditions [106]. These advancements highlight the potential of surface-engineered membranes in achieving robust anti-wetting performance and stable long-term operation in practical applications.

From an engineering perspective, additional limitations include pressure drop, energy consumption, and membrane lifetime. Gabelman and Hwang [107] reported that increasing module packing density improves mass transfer efficiency but simultaneously increases hydraulic resistance and flow maldistribution, leading to higher pumping energy requirements. Moreover, membrane degradation caused by prolonged exposure to oxidants, thermal stress, and chemical cleaning agents indicates that membrane lifetime remains a key uncertainty in scale-up applications. In addition, vacuum systems or sweep-gas supply are necessary to maintain the driving force for oxygen removal, and their energy consumption should be considered in process evaluation, particularly when ultra-low dissolved oxygen concentrations are required [108]. Techno-economic analyses have shown that membrane deaeration systems offer advantages such as compact design, modular operation, rapid start-up, and reduced steam consumption compared to conventional thermal deaerators [109,110,111]. However, their economic competitiveness in large-scale boiler feedwater applications remains uncertain due to membrane replacement costs, cleaning frequency, and auxiliary energy consumption associated with vacuum or gas supply systems. Gong et al. [112] conducted an application study of membrane deaerator through the case of thermal recovery boiler in the Bohai K oilfield (processing capacity of 60 t/h). In this case, the membrane deoxygenation system occupies only 50 m^2^ (thermal deoxygenation 80 m^3^) and has an operating weight of 45 t (thermal deoxygenation 132 t). It also achieves the removal effect of dissolved oxygen of ≤0.02 mg/L under normal temperature operation, which proves the lightweight advantage of the technology in the space-constrained scenario of offshore platforms. Gao [113] studied the application of a degassing membrane in boiler feedwater deoxygenation through a case study of a 330 MW heating unit. The degassing membrane device of the unit had a processing capacity of 100 ton/h, and the dissolved oxygen was reduced from 9550 μg/L to below 100 μg/L, with a removal rate of 98.96%, and the condenser vacuum was significantly improved (94.72 kPa). Yang et al. [114] verified the engineering applicability of degassing membrane technology through a case study of boiler water deoxygenation in a waste incineration power plant. In a medium-scale study, three degassing membranes in series were used to treat 5 m^3^/h of demineralized water. Under the conditions of 20 °C, −0.1 MPa vacuum and high-purity nitrogen purging, the dissolved oxygen was reduced from 8 mg/L to 7 μg/L, which was significantly better than the boiler feedwater standard of 15 μg/L.

**Figure 8:**
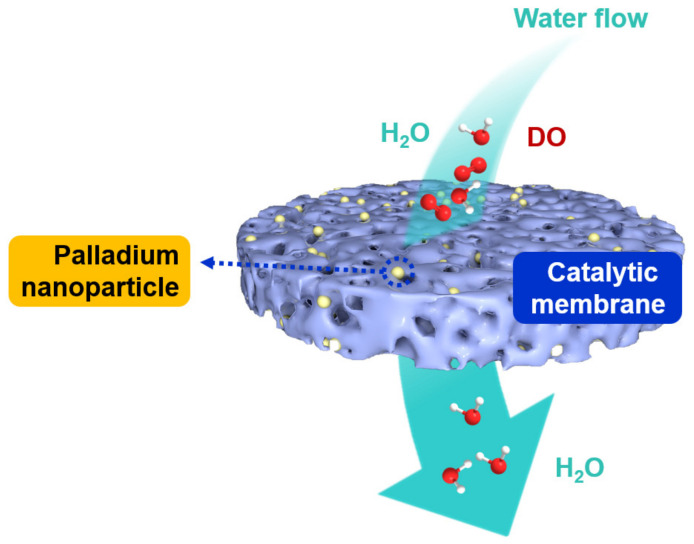
The catalytic membrane used for the chemical removal of dissolved oxygen from water.

In addition, membrane deoxygenation technology has also been successfully extended to other important industrial fields. Zegeye et al. [115] developed an integrated membrane distillation-selective electrodialysis system for sustainable lithium recovery from geothermal brine. In parallel, the use of hollow fiber membrane contactors (HFMCs) has shown significant promise in gas–liquid mass transfer processes, particularly for CO_2_ desorption. Vadillo et al. [116] highlight the advantages of HFMCs over conventional desorption columns, including higher interfacial area, enhanced mass transfer coefficients, and more compact system design, and underscore the importance of membrane surface engineering to prevent wetting and maintain long-term operational stability in CO_2_ capture systems. Zhou et al. [117] conducted an experimental study on the removal of hydrochloric acid using hollow fiber membrane degassing technology. They systematically investigated the effects of membrane area, feed flow rate and concentration on the removal efficiency. Under the conditions of membrane area of 1.0 m^2^, flow rate of 0.12 m/min and vacuum degree of 0.090 MPa, the removal rate of 6 mol/L hydrochloric acid was as high as 85%. Feng [118] studied the process efficiency of CO_2_ removal by a degassing membrane through the case of semiconductor ultrapure water preparation. Compared with the traditional NaOH dosing system, the degassing membrane (two parallel and two series design) stabilized the conductivity of the secondary reverse osmosis (RO) effluent at 1.29–2.21 μS/cm, providing cross-domain technical verification for the boiler feedwater deoxygenation process. Lee et al. [119] systematically studied the effect of using a hollow fiber membrane for deoxygenation of feedwater and return water in district heating systems and its inhibitory effect on steel pipe corrosion on a pilot scale, and reduced the dissolved oxygen concentration of the effluent to below 10 μg/L. After deoxygenation treatment, the corrosion weight loss of the steel pipe sample was significantly reduced, and the corrosion resistance was improved by inhibiting the cathodic oxygen reduction reaction. In addition to physical deoxygenation based on gas–liquid balance and membrane contact to enhance mass transfer, researchers are also actively exploring new catalytic deoxygenation pathways that combine efficient chemical reactions with membrane separation processes. The core idea is to load catalytically active components into membrane materials or membrane modules so that dissolved oxygen is rapidly catalytically reduced while mass transfer is enhanced, thereby achieving faster removal. Vaart et al. [120] studied commercial membrane contactors with in situ deposited palladium catalysts for dissolved oxygen removal in water. By successfully coating Pd catalysts on PP hollow fiber membranes, the feasibility of deoxygenation with hydrophobicity after membrane surface modification was verified. As shown in Figure 8, Zhang et al. [121] prepared polymethacrylate hybrid microgels (PMAA-Pd) and introduced them into the pores of a highly asymmetric polyether sulfone (PES) microfiltration membrane, where they reversibly expanded in response to external stimuli and achieved efficient catalysis by utilizing the high dispersion of palladium nanoparticles. At room temperature, with a residence time of 32.3 s, the three-layer membrane can reduce the dissolved oxygen concentration to 10 ppb and has good stability. By functionalizing membrane materials and coupling physical separation with chemical reactions, it is hoped that the limitations of physical mass transfer processes in terms of kinetics and thermodynamics can be overcome, opening up new paths for achieving ultra-low dissolved oxygen concentration control and adapting to more complex water quality or harsh conditions such as low temperature and low pressure.

## 4. Summary and Outlook

Boiler feedwater deoxygenation is a crucial step in ensuring the safe and reliable operation of thermal systems. While traditional physical deoxygenation technologies (thermal deoxygenation, analytical deoxygenation, and vacuum deoxygenation) have matured in engineering practice, they are limited by thermodynamic equilibrium and macroscopic gas–liquid contact area, making it difficult to achieve deep deoxygenation at the ppb level with low energy consumption. Furthermore, they still suffer from high energy consumption, complex equipment, and limited system adaptability. Against this backdrop, membrane methods utilize hydrophobic porous membranes as highly efficient and stable gas–liquid interfaces, significantly increasing the interphase contact surface area and decoupling fluid flow from interphase mass transfer, thus reshaping the dissolved oxygen removal mechanism from a kinetic perspective. Their advantages include compact equipment, low energy consumption, and high modularity, and their feasibility has been verified in multiple industrial scenarios. Currently, boiler feedwater deoxygenation technology is evolving along a “mass transfer enhancement” path: industrial applications are transitioning from traditional physical methods to membrane methods, and research is focusing on functionalizing membrane materials, optimizing membrane module structures, and catalytic coupling to overcome engineering bottlenecks such as membrane fouling control, pressure drop optimization, and system durability. Overall, with the further development of membrane materials science and mass transfer theory, membrane deoxygenation is expected to achieve more efficient and economical deep deoxygenation under higher parameters and more complex water quality conditions.

## Figures and Tables

**Table 1:** The corrosion galvanic cell reactions of boilers.

Anodic reaction	$M-ne^{-}\;\to {\;M}^{n+}$	$E^{{}^{\circ}}=-0.409\;V\;(M^{2+}={\mathrm{Fe}}^{2+})$
Cathodic reaction	$O_{2}+2H_{2}O+4e^{-}\;\to \;4{\mathrm{OH}}^{-}$	$E^{{}^{\circ}}=+0.401\;V$
Overall reaction	$nM+O_{2}+2H_{2}O\;\to {\;M(\mathrm{OH})}_{n}$	$E_{\mathrm{cell}}^{{}^{\circ}}=0.810\;V\;(M^{n+}={\mathrm{Fe}}^{2+})$

**Table 2:** Comparison of thermal deaerators and outlet dissolved oxygen performance.

Deaerator Type	Outlet DO Concentration	Key Innovations and Performance Highlights	Ref.
High-Pressure Rotating Film	Less than 7 μg/L	Increased gas–liquid contact area, reduced vibration and noise	[39]
Low-Temperature Rotating Film	Less than 10 μg/L	Ambient inlet temperature, no auxiliary heating required	[40]
Thermal Rotating Film	Less than 6.3 μg/L	Forms water film skirt through rotating film tubes for deep deoxygenation, effectively recovers moisture in exhaust	[41]
Nitrogen Sweep Thermal	Less than 7 ppb	Nitrogen sweep reduces O_2_ to <7 ppb and CO_2_ to undetectable	[42]
Thermal Deaerator Method	Less than 7 ppb	Preferred embodiments achieve <~7 ppb through heating and stripping	[43]
Deoxidizing Condenser	Less than 7 ppb	Ensures oxygen content in condensate < 7 ppb	[44]
Polypropylene Spheres Assisted	Less than 7 ppb	System-level thermal deaeration and re-oxygenation suppression architecture by polypropylene	[45]

**Table 3:** The comparison between traditional methods and membrane methods.

Technology	Operating Temp.	Achievable DO Level	Energy Demand	Advantages	Limitations
Thermal	High (90–180 °C)	Low (ppb level)	High	Simple principle, mature tech	High energy cost, scaling issues, not for heat-sensitive fluids
Stripping /Desorption	Ambient (~60 °C)	Moderate (ppb level)	Moderate	Lower temp than thermal, effective	Requires carrier gas (N_2_), risk of gas dissolution, evaporation loss
Vacuum	Ambient (~80 °C)	Moderate (ppb level)	Moderate-low	No carrier gas needed, simple	Requires vacuum pump, limited by vapor pressure, evaporation
Membrane	Ambient (5~40 °C)	Very Low (<0.1 ppb)	Low-medium	No phase mixing, compact, high efficiency, modular	Membrane wetting risk, pore clogging, module cost
Catalytic Membrane	Ambient (20~60 °C)	Moderate (ppb level)	Low	Fast kinetics, works at low conc.	Catalyst cost (Pd), catalyst poisoning risk, complex fabrication

**Table 4:** The comparison of different membrane materials and corresponding properties.

Membrane Material	Typical Module Type	Deoxygenation Efficiency	Mass Transfer Coefficient (k_m_)	Operating Conditions	Stability and Comments
PP (Polypropylene)	Hollow fiber	Very high (removal > 99%)	High (10^−5^~10^−4^)	Vacuum or N_2_ sweep	Excellent; low cost, widely used commercially, but lower mechanical strength.
PVDF (Polyvinylidene Fluoride)	Hollow fiber	High (removal > 95%)		Vacuum or sweep	Good; high mechanical strength, resistant to ozone/oxidation compared to PP.
PTFE (Polytetrafluoroethylene)	Hollow fiber	High (removal > 99.9%)	Moderate	Vacuum or sweep	Outstanding; highest chemical/thermal resistance; maintains non-wetting state under harsh conditions.
Ceramic (Al_2_O_3_)	Tubular/hollow fiber	High	Moderate (varies with pore size)	Vacuum or sweep	Excellent; inert, durable, but brittle and high cost; suitable for aggressive environments.
Silicone Rubber (PDMS)	Hollow fiber	Moderate	Very high (10^−4^~10^−3^)	Vacuum	Low; prone to wetting; low hydrophobicity retention; mainly used in specific catalytic deoxygenation.

## Data Availability

No new data were created or analyzed in this study. Data sharing is not applicable to this article.

## Abbreviations

The following abbreviations are used in this manuscript:

DO	Dissolved oxygen
PP	Polypropylene
PDMS/PP	Polydimethylsiloxane/polypropylene
PVDF	Polyvinylidene fluoride
PMAA-Pd	polymethacrylate hybrid microgels
PES	polyether sulfone
PTFE	Polytetrafluoroethylene
HFMC	Hollow fiber membrane contactor
LEP	Liquid entry pressure
